# Enterohemorrhagic *Escherichia coli* O157∶H7 Gene Expression Profiling in Response to Growth in the Presence of Host Epithelia

**DOI:** 10.1371/journal.pone.0004889

**Published:** 2009-03-18

**Authors:** Narveen Jandu, Nathan K. L. Ho, Kevin A. Donato, Mohamed A. Karmali, Mariola Mascarenhas, Simon P. Duffy, Chetankumar Tailor, Philip M. Sherman

**Affiliations:** 1 Department of Pathology, Stanford University School of Medicine, Stanford, California, United States of America; 2 Research Institute, Hospital for Sick Children, University of Toronto, Toronto, Ontario, Canada; 3 Laboratory of Foodborne Zoonosis, Public Health Agency of Canada, Guelph, Ontario, Canada; University of Würzburg, Germany

## Abstract

**Background:**

The pathogenesis of enterohemorrhagic *Escherichia coli* (EHEC) O157∶H7 infection is attributed to virulence factors encoded on multiple pathogenicity islands. Previous studies have shown that EHEC O157∶H7 modulates host cell signal transduction cascades, independent of toxins and rearrangement of the cytoskeleton. However, the virulence factors and mechanisms responsible for EHEC-mediated subversion of signal transduction remain to be determined. Therefore, the purpose of this study was to first identify differentially regulated genes in response to EHEC O157∶H7 grown in the presence of epithelial cells, compared to growth in the absence of epithelial cells (that is, growth in minimal essential tissue culture medium alone, minimal essential tissue culture medium in the presence of 5% CO_2_, and Penassay broth alone) and, second, to identify EHEC virulence factors responsible for pathogen modulation of host cell signal transduction.

**Methodology/Principal Findings:**

Overnight cultures of EHEC O157∶H7 were incubated for 6 hr at 37°C in the presence or absence of confluent epithelial (HEp-2) cells. Total RNA was then extracted and used for microarray analyses (Affymetrix *E. coli* Genome 2.0 gene chips). Relative to bacteria grown in each of the other conditions, EHEC O157∶H7 cultured in the presence of cultured epithelial cells displayed a distinct gene-expression profile. A 2.0-fold increase in the expression of 71 genes and a 2.0-fold decrease in expression of 60 other genes were identified in EHEC O157∶H7 grown in the presence of epithelial cells, compared to bacteria grown in media alone.

**Conclusion/Significance:**

Microarray analyses and gene deletion identified a protease on O-island 50, gene Z1787, as a potential virulence factor responsible for mediating EHEC inhibition of the interferon (IFN)-γ-Jak1,2-STAT-1 signal transduction cascade. Up-regulated genes provide novel targets for use in developing strategies to interrupt the infectious process.

## Introduction

Enterohemorrhagic *Escherichia coli* (EHEC; also variously referred to as Verocytotoxin producing *E. coli*, VTEC and Shiga toxin producing *E. coli*, STEC), serotype O157∶H7 is a serious human enteric pathogen, as evidenced by an outbreak across North America due to the ingestion of contaminated spinach [1] . Symptoms of EHEC infection range from mild diarrhea to hemorrhagic colitis and, in the most severe cases, the hemolytic uremic syndrome [2]. EHEC O157∶H7 virulence factors include phage-encoded Shiga toxins and intimate bacterial adhesion, described as attaching-effacing lesions [3]. Formation of attaching-effacing lesions is caused by effector proteins and a type three secretion system, which are encoded on a locus of enterocyte effacement (LEE) pathogenicity island [4]. EHEC O157∶H7 disease pathogenesis also is related to microbial modulation of eukaryotic signal transduction cascades. For instance, previous studies showed that EHEC disrupts host innate immune signaling responses, including the IFNγ-Jak1,2-STAT-1 signal transduction cascade [5]–[7]. EHEC subversion of STAT-1 activation by IFNγ involves a prokaryotic factor that requires bacterial contact with the host epithelial cell.

The complete genome sequences of two EHEC O157∶H7 strains have been reported previously [8], [9]. *E. coli* O157∶H7 strain EDL933 contains 1.34 Mb of genomic information that is absent in non-pathogenic *E. coli*, strain K-12 [9]. This extra DNA is organized into 177 putative genomic islands, ranging in size from less than 10 to greater than 100 kilobases [8]. Global gene expression profiling is a strategy that can be used to better understand mechanisms underlying host pathogen interactions in response to infection (reviewed in: [10]). Studies describing gene expression profiling of *E. coli* are limited having focused on either phylogenetic analyses [11]–[14] or changes in gene expression in response to varying environmental growth conditions [15]–[20]. By contrast, only a few studies have delineated *E. coli* gene expression in the context of infection models. One recent study performed gene expression profiling of two urinary tract *E. coli* strains, originally isolated from subjects with asymptomatic bacteriuria [21]. Only one previous study described a differential gene expression pattern of EHEC O157∶H7 during pathogen growth with erythrocytes, relative to growth in tissue culture medium [22]. Consequently, gene expression studies of EHEC O157∶H7 in the context of infected epithelia are lacking. In addition, despite extensive research on the effects of EHEC on host cell signaling, relatively few bacterial factors responsible for pathogenic effects have been identified [23].

Therefore, the purpose of this study was to perform comparative gene expression analysis of EHEC O157∶H7 grown in the presence of cultured epithelial cells, relative to growth in the absence of epithelial cells. In addition, gene expression analysis was performed to identify virulence factors involved in EHEC disruption of the IFNγ-Jak1,2-STAT-1 signal transduction cascade.

## Results

### Relatedness of gene-expression profiles of EHEC O157∶H7, strain CL56 cultured under four different growth conditions

Microarray analysis was employed to identify global gene expression changes of EHEC O157∶H7 grown in the presence of epithelial cells, relative to the same organism grown in the absence of epithelial cells (Penassay broth, minimal essential tissue culture medium or minimal essential tissue culture medium in 5% CO_2_). Principle Components Analysis (PCA), using the Partek Genomics Suite, was performed to confirm the relatedness of data sets. This method of cluster analysis also was used to identify outliers in the data set. Data from all 19 microarray gene chips generated a correlation value (i.e. PCA value) of 68.9% with two distinct outliers. Removal of these outliers resulted in a correlation value of 68.4% for the remaining 17 gene chips, which were then used for all subsequent data analyses (**Supplemental** **Figure S1**). Individual principle components analysis for each group of gene chips, representing EHEC growth under all four conditions each revealed correlation values of 100%.

### Gene expression pattern of EHEC O157∶H7 housekeeping genes under different growth conditions

**Figure 1:**
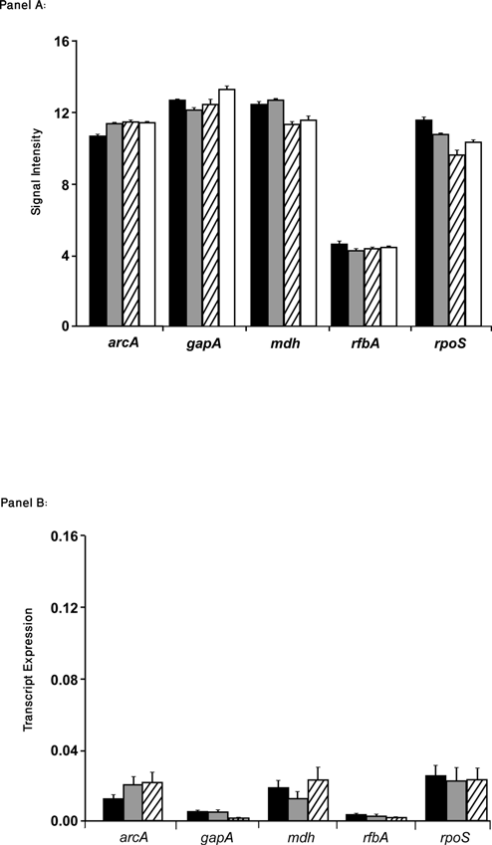
Relative expression of EHEC O157∶H7, strain CL56 housekeeping genes in response to different growth conditions. [Panel A] Signal intensities of housekeeping genes (*arcA*, *gapA*, *mdh*, *rfbA* and *rpoS*) from microarray chips in response to EHEC O157∶H7 grown under four different conditions. All housekeeping genes showed similar levels of expression, irrespective of the bacterial growth condition. Data are presented as means±SEM. [Panel B] qRT-PCR of the same housekeeping genes confirms comparable levels of transcript expression, irrespective of EHEC O157∶H7 growth condition. Data points from triplicates of EHEC O157∶H7 grown in contact with epithelial cells, microbial growth in minimal essential medium (+/−5% CO_2_) were extrapolated from a standard curve generated for each primer pair using samples of the same bacterial strain grown in Penassay broth alone. As described in the *Experimental procedures*, data analysis was performed using the 7500 Sequence Detection System Software package (Applied Biosystems). Black bars represent EHEC O157∶ H7 grown in the presence of epithelial cells; grey bars: pathogen grown in minimal essential medium in atmospheric conditions or in 5% CO_2_ (stripped bars). EHEC grown in Penassay broth are shown in white bars [Panel A].

Housekeeping genes provided internal controls, which were used as a measure of mRNA expression levels irrespective of growth condition [24]. The expression patterns of five *E. coli* housekeeping genes were determined including, *arcA*, *gapA*, *mdh*, *rfbA*, and *rpoS* [25] during EHEC O157∶H7 growth in the presence or absence of epithelial cells. The signal intensities of these select housekeeping genes, as determined by microarray analysis, are shown in **Figure 1** **, Panel A**. Signal intensities of these housekeeping genes were comparable for samples of EHEC O157∶H7 under each of the four different growth conditions.

As shown in **Figure 1** **, Panel B**, qRT-PCR of the housekeeping genes, *arcA*, *gapA*, *mdh*, *rfbA*, and *rpoS* also showed comparable levels of transcript expression (n = 3, p>0.05, ANOVA), irrespective of EHEC growth condition. Collectively, these results validate the microarray data and allowed for subsequent data analysis of expression patterns for selected potential virulence genes.

### Differentially regulated genes during EHEC O157∶H7 growth in the presence of epithelial cells, relative to the absence of epithelial cells

**Figure 2:**
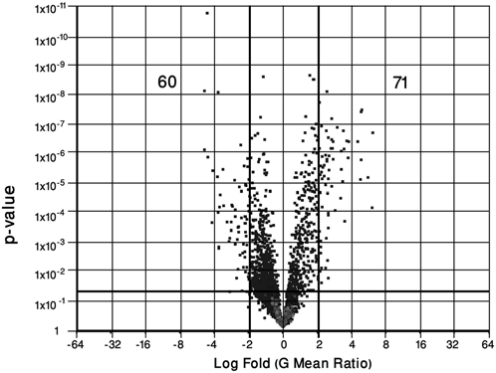
Volcano plot showing differential expression of EHEC O157∶H7, strain CL56 genes during bacterial growth in the presence of epithelial cells, relative to the absence of epithelial cells. Data points were extracted from a 1-way analysis of variance (ANOVA): Gene expression changes during EHEC growth in the presence of cultured epithelial cells, relative to pathogen growth in the absence of epithelial cells. The x-axis represents ‘log fold change’ and the corresponding dark vertical lines represent cut-offs at log_2.0_-fold decreases and increases. The y-axis represents p-values and the corresponding dark horizontal lines indicate a p value cut-off of 0.05. Values presented represent the number of down- and up-regulated genes, respectively.

Differentially regulated genes in response to EHEC O157∶H7 growth in the presence of epithelial cells relative to the absence of HEp-2 cells, were identified by performing a 1-way ANOVA on the gene chip data set. As described in the *Materials and Method* section, a 2.0 log fold change and a *p* value of 0.05 were employed as cut-off values. These statistical parameters identified 131 genes (60 down-regulated and 71 up-regulated) that were differentially regulated in EHEC due to growth in the presence of HEp-2 cells, relative to the absence of epithelial cells **Figure 2**. (Individual comparisons may be found in **Supplemental** **Figure S2** and the corresponding lists of genes can be found in **Supplemental** **Tables S1** **,** **S2** **and** **S3**).

### Functional classification of differentially regulated genes in EHEC O157∶H7 grown in the presence or absence of eukaryotic cells

**Figure 3:**
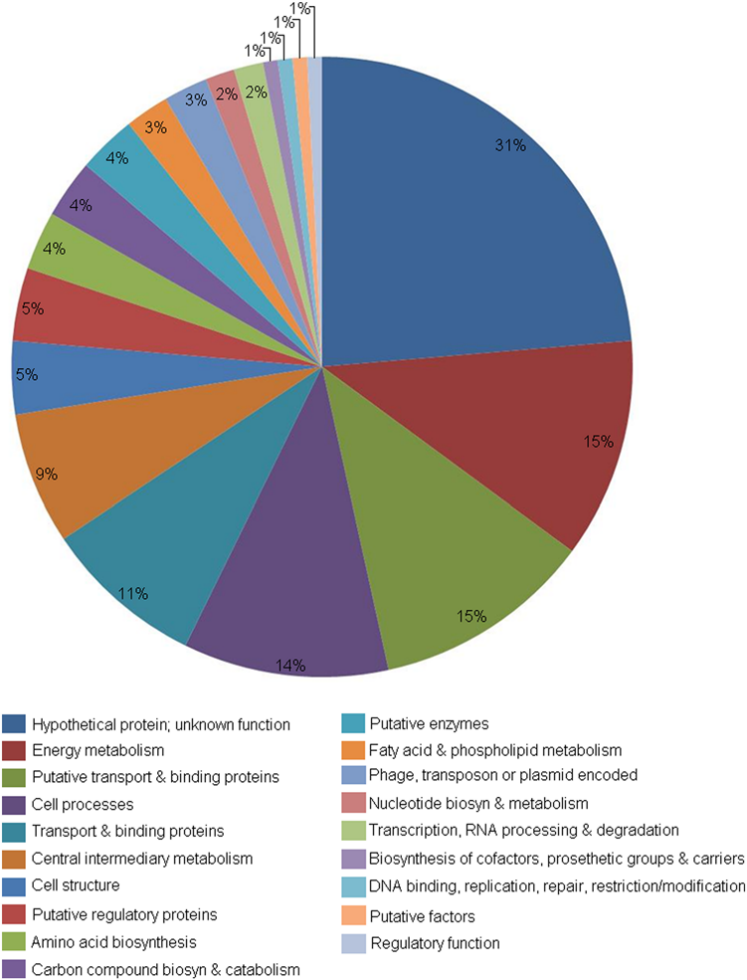
Functional classification of the 131 differentially regulated genes during EHEC O157∶H7 growth in the presence of epithelial cells, relative to the absence of epithelial cells, as summarized in a pie chart [27]. Each pie slice represents a major functional group of genes. Numbers shown represent percentage.

Differentially regulated genes were categorized using a previously described functional classification system [21], [26]. As shown in **Figure 3** [27], the majority of the gene changes were clustered in the functional group comprising genes which encode for transport and binding proteins (8.4%), energy metabolism factors (11.5%), central intermediary metabolism factors (6.9%), and factors involved in cellular processes (10.7%). The next set of functional groups displaying the greatest gene expression changes in relation to EHEC O157∶H7 growth conditions were those encoding macromolecule biosynthesis (amino acid biosynthesis: 3.5%; biosynthesis of cofactors: 0.8%; carbon compound biosynthesis: 3.1%), cell structure components (3.8%), regulatory components (0.8%), and putative factors (0.8–11.5%). As in most microarrays, hypothetical proteins or genes of unknown function accounted for the greatest percentage (23.7%) of all differentially regulated genes in each comparison.

### Hierarchical clustering of EHEC O157∶H7 up-regulated genes in response to growth in the presence of epithelial cells

**Figure 4:**
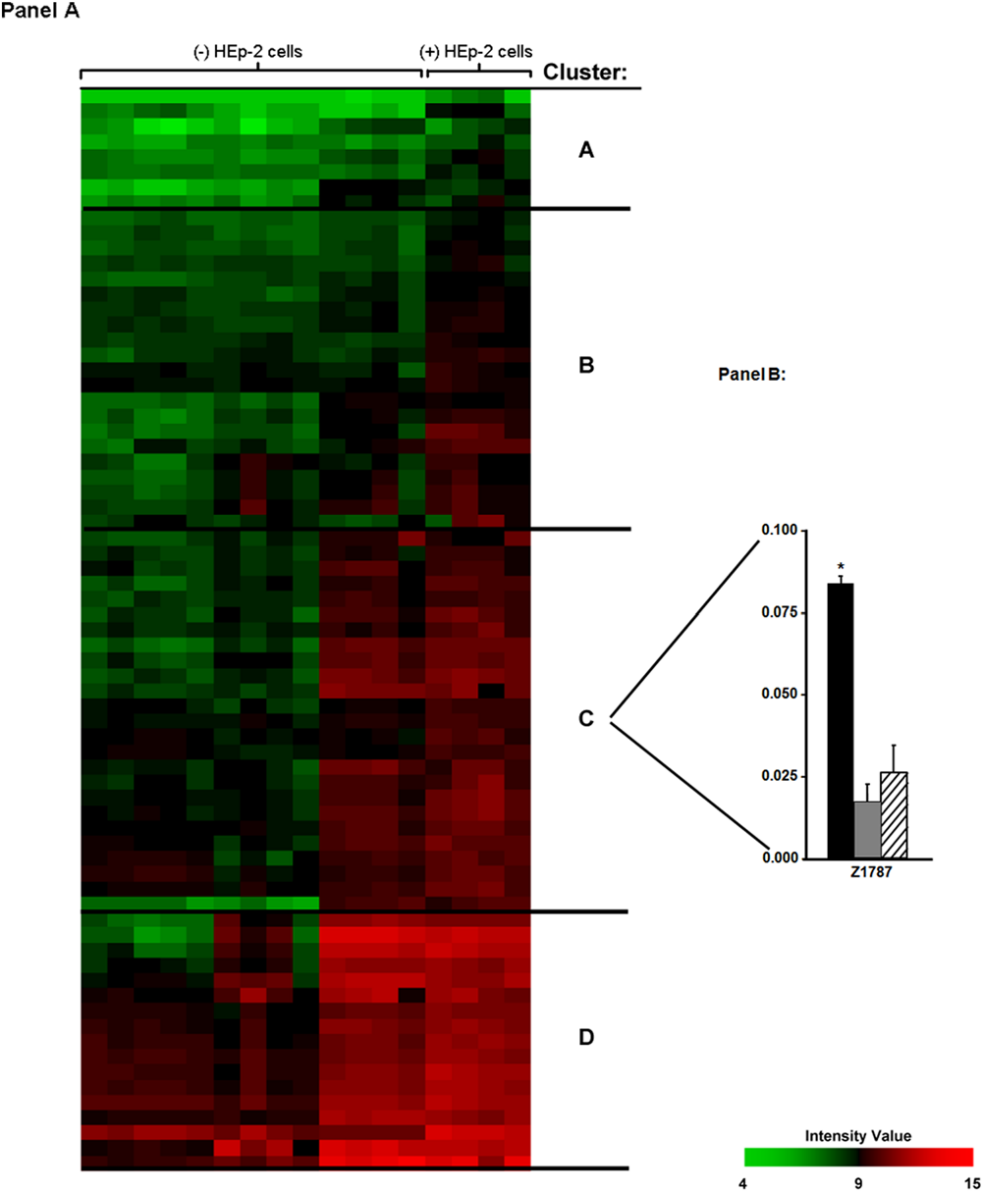
Hierarchical cluster plot showing relative expression patterns of EHEC O157∶H7 up-regulated genes in the presence of epithelial cells, relative to the absence of epithelial cells [Panel A]. Horizontal rows represent distinct genes and vertical columns represent individual samples of EHEC O157∶H7 grown in the presence of epithelial cells [(+) HEp-2], relative to the absence of epithelial cells [(−) HEp-2]. Relative expression patterns of EHEC O157∶H7, strain CL56 gene Z1787 [Panel B]. qRT-PCR of Z1787 showing differences in transcript expression for EHEC O157∶H7 under varying growth condition. Data points are derived from triplicates of EHEC O157∶H7 grown in epithelial cells versus microbial growth in minimal essential medium (−/+5% CO_2_) were extrapolated from a standard curve generated for each primer pair using EHEC O157∶H7 grown in Penassay broth. Data analysis was performed using the 7500 Sequence Detection System Software package (Applied Biosystems). Black bars represent EHEC O157∶ H7 grown in the presence of HEp-2 cells; grey bars represent the pathogen grown in minimal essential medium in room air; stripped bars represent microbial growth in minimal essential medium in 5% CO_2_.

Cluster analysis was subsequently performed to identify the relative expression pattern of up-regulated genes for EHEC O157∶H7 grown in the presence of epithelial cells, compared with the pathogen grown in the absence of host epithelia. Hierarchical clustering was employed to reveal degrees of similarity in the gene expression profiles for the 71 up-regulated genes in EHEC O157∶H7 grown in the presence of HEp-2 cells, relative to pathogen growth in the absence of eukaryotic cells. As shown in **Figure 4** **, Panel A**, up-regulated genes were grouped into four major clusters: labeled A–D. Genes in clusters A–D are listed in **Table 1**. Genes in Cluster A primarily encode metabolism factors and enzymes. In Cluster B, multiple genes coding for the *E. coli* type three secretion system apparatus (i.e. *escT*, *escS*, *escU*, and *escR*) were up-regulated. In cluster C, genes responsible for lipopolysaccharide biosynthesis (i.e. *wbdP*, *wzx*, and *wbdO*) were up-regulated. In addition, in Cluster C, a lipoprotein (*rlpB*), a prolipoprotein signal peptidase (*lspA*), and a putative receptor (Z1178) were highly up-regulated as a result of pathogen growth in the presence of epithelial cells. Cluster D contained several transport factors (i.e. *chuA*, *chuS*, and *chuT*).

**Table 1:** Differentially up-regulated genes corresponding to Clusters A–D, FIGURE 4.

Probeset ID:	Gene	Gene product / functional description	Fold change
**Cluster A: 8 up**			
1767702_s_at	*ilvC*	ketol-acid reductoisomerase	4.8518
1761586_s_at	*leuB*	3-isopropylmalate dehydrogenase	3.3419
1768228_s_at	*actP*	acetate permease	2.6979
1767691_s_at	*gltB*	glutamate synthase, large subunit	2.6953
1764907_s_at	*betB*	NAD+-dependent betaine aldehyde dehydrogenase	2.3903
1767859_s_at	*ordL*	probable oxidoreductase	2.3018
**Cluster B: 21 up**			
1761408_s_at	*entD*	enterobactin synthetase component D	6.1178
1762165_s_at	*bioC*	biotin biosynthesis; reaction prior to pimeloyl CoA	4.7654
1766687_s_at	Z4919	putative ATP-binding protein of ABC transport system	3.6493
1763215_s_at	*bioD*	dethiobiotin synthetase	3.0056
1767427_s_at	Z4853	putative acyl carrier protein	2.5431
1764491_s_at	*escT*	escT	2.4659
1765592_s_at	*urge*	putative urease accessory protein G	2.4320
1759270_s_at	*gltD*	glutamate synthase, small subunit	2.4262
1769074_s_at	*fepC*	ATP-binding component of ferric enterobactin transport	2.4050
1761897_s_at	Z4854	putative acyl carrier protein	2.2304
1767820_s_at	*dniR*	transcriptional regulator for nitrite reductase	2.2142
1763082_s_at	*dppD*	putative ATP-binding component	2.1920
1763133_s_at	*escS*	escS	2.1702
1767609_s_at	*escU*	escU	2.1476
1767451_s_at	*gnd*	gluconate-6-phosphate dehydrogenase, decarboxylating	2.1268
1764551_s_at	*ureF*	putative urease accessory protein F	2.1104
1762528_s_at	*escR*	escR	2.0480
1760850_s_at	*waaY*	putative LPS biosynthesis protein	2.0172
**Cluster C: 25 up**			
1759518_s_at	*exbB*	uptake of enterochelin	2.1552
1760472_s_at	Z1341	unknown protein encoded by prophage CP-933M	2.5132
1760730_s_at	*chuU*	putative permease of iron compound ABC transport	3.8076
1760999_s_at	*wbdP*	glycosyl transferase	2.7773
1761010_s_at	*beta*	choline dehydrogenase, a flavoprotein	3.3830
1761172_s_at	*terE*	putative phage inhibition, colicin resis & tellurite resis.	2.5218
1761302_s_at	*terC*	putative phage inhibition, colicin resis & tellurite resis	3.4412
1762953_s_at	*wzx*	O antigen flippase Wzx	2.1944
1763001_s_at	*terE*	putative phage inhibition, colicin resis & tellurite resis	3.2745
1764750_s_at	*rlpB*	A minor lipoprotein	2.2008
1764929_s_at	*nuoG*	NADH dehydrogenase I chain G	2.3666
1765359_s_at	*chuW*	putative oxygen independent coproporphyrinogen III oxidase	4.9399
1765620_s_at	*glyA*	serine hydroxymethyltransferase	2.0473
1765959_s_at	*lspA*	prolipoprotein signal peptidase (SPase II)	2.3639
1766483_s_at	Z1178	putative receptor	4.5374
1766799_s_at	*yafH*	putative acyl-CoA dehydrogenase (EC 1.3.99.-)	2.5048
1766809_s_at	Z2778	putative aldehyde dehydrogenase	2.0451
1766905_s_at	*terA*	putative phage inhibition, colicin resis & tellurite resis	2.8857
1767319_s_at	*acrB*	acridine efflux pump	2.3176
1767505_s_at	*entE*	2,3-dihydroxybenzoate-AMP ligase	3.7641
1767555_s_at	Z1787	unknown protein encoded by prophage CP-933N	2.6642
1768558_s_at	*wbdO*	glycosyl transferase	2.7212
**Cluster D: 17 up**			
1762260_s_at	*chuA*	outer membrane hemehemoglobin receptor	6.0326
1763313_s_at	*chuS*	putative hemehemoglobin transport protein	5.5553
1768770_s_at	*chuT*	putative periplasmic binding protein	3.4813
1763337_s_at	*entF*	ATP-dependent serine activating enzyme	3.2049
1760675_s_at	*per*	perosamine synthetase	2.7632
1764806_s_at	*wbdQ*	GDP-mannose mannosylhydrolase	2.6713
1766849_s_at	*wzy*	O antigen polymerase	2.5446
1766583_s_at	*aceF*	pyruvate dehydrogenase	2.5072
1759808_s_at	*fadB*	4-enzyme protein	2.4949
1761631_s_at	*terD*	putative phage inhibition, colicin resis & tellurite resis	2.4640
1759686_s_at	*fcI*	fucose synthetase	2.4502
1766456_s_at	Z3198	GDP-mannose dehydratase	2.2236
1764835_s_at	Z5136	Hypothetical protein / unknown function	2.1700
1759443_s_at	*manC*	mannose-1-P guanosyltransferase	2.1422
1764320_s_at	*gltA*	citrate synthase	2.0958

### Gene Z1787: a novel putative virulence factor involved in EHEC O157∶H7 disruption of IFNγ-Jak1,2-STAT-1 signaling

To confirm the microarray data, qRT-PCR was performed. As shown in **Figure 4** **, Panel B**, transcript expression of gene Z1787 was significantly greater following EHEC growth in the presence of epithelial cells than when the bacterium was grown in the absence of epithelial cells (n = 3; ANOVA, p<0.001).

**Figure 5:**
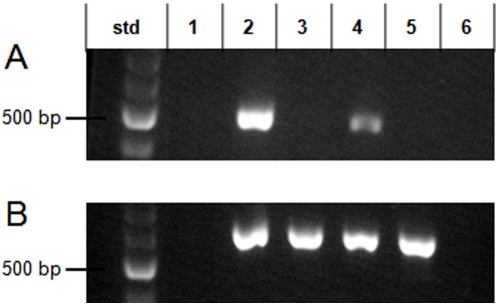
Transcript expression of EHEC O157∶H7 gene Z1787 in wild-type and mutant strains. As described in the *Experimental procedures*, gene deletion was performed using the Lambda Red technique [27]–[28]. Isogenic mutants were then screened and verified using reverse transcriptase polymerase chain reaction (RT-PCR). PCR products were electrophoresed on 2% agarose gel, stained with ethidium bromide, and bands then visualized under a UV lamp. The standard employed was a 100 bp ladder. Lane 1: negative water control; Lane 2: EHEC strain CL56; Lane 3: EPEC strain E2348/69; Lane 4: EHEC strain EDL 933 (parent to the mutant); Lane 5: ΔZ1787; Lane 6: no transcript control. [Panel A] transcript expression of EHEC gene Z1787; [Panel B] transcript expression of EHEC *gapA*. Primers are described in Table 2 of the *Materials and Methods*.

Subsequently, isogenic knock-out mutants were generated by employing the Lambda Red technique for gene deletion, as previously described [28], [29]. Gene Z1787 was selected as a candidate virulence factor to be investigated via mutational analysis due to its location on genomic island OI-50, a >100 kbp genomic island. Gene knock-outs were verified using reverse transcriptase polymerase chain reaction (RT-PCR). As shown in **Figure 5**, transcript expression for EHEC gene Z1787 was positive in strain CL56 and parent strain EDL933, but absent in both the knock-out, Δ*Z1787* and in EPEC strain E2348/69.

**Figure 6:**
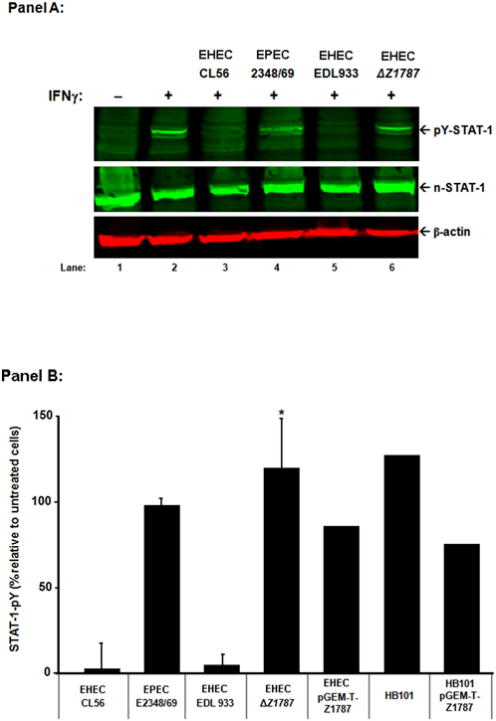
EHEC O157∶H7 gene Z1787 is a virulence factor responsible for inhibition of STAT-1 tyrosine phosphorylation. Cultured epithelial cells were infected with wild-type EHEC O157∶H7, strains CL56 and strain EDL 933, EHEC ΔZ1787 (in strain EDL 933) and EPEC O127∶H6 (MOI 100∶1) for 6 hr (or as described in the *Experimental procedures*) at 37°C in 5% CO_2_. Washed cells were then stimulated with interferon (IFN)-γ (50 ng/mL) for 0.5 hr at 37°C in 5% CO_2_. Whole cell protein extracts were collected and immunoblots probed with either anti-latent-STAT-1 or anti-phospho-STAT-1 and anti-β-actin primary antibodies, followed by respective secondary antibodies. [Panel A] Positively staining bands were detected by using an infrared scanner. Lanes 1 & 2: uninfected epithelial cells in the absence and presence of interferon-γ, respectively; Lane 3: EHEC O157∶H7, strain CL56 inhibited STAT-1 tyrosine phosphorylation; Lanes 4: EPEC did not disrupt STAT-1 tyrosine phosphorylation. Lane 5: Wild-type EHEC O157∶H7, strain EDL933 inhibited IFNγ stimulated STAT-1 activation. Lane 6: Gene disruption of Z1787 in EDL 933 prevented EHEC subversion of STAT-1 signaling in response to IFNγ. [Panel B] Densitometry of positively stained bands was quantified using software imbedded in the infrared scanner. Quantification of STAT-1 tyrosine phosphorylation in epithelial cells infected with EHEC O157∶H7 complemented with gene Z1787 using the pGEM-T vector also was determined. As a positive control, levels of STAT-1 tyrosine phosphorylation also were determined in epithelial cells treated with non-pathogenic *E. coli* strain HB101: wild-type bacteria did not inhibit STAT-1 activation, while HB101+gene Z1787 (inserted on the pGEM-T vector) resulted in partially reduced levels of STAT-1 tyrosine phosphorylation (n = 1–4; ANOVA, *p<0.01).

Phenotypic analysis of the isogenic mutants was performed by employing an *in vitro* tissue culture infection model followed by immunoblotting. As shown in **Figure 6** **, Panel A**, unlike the parent strain, EHEC strain EDL933, Δ*Z1787* did not block STAT-1 tyrosine phosphorylation in response to IFNγ stimulation. Quantification of immunoblots (**Figure 6** **, Panel B**) demonstrate significantly greater STAT-1 tyrosine phosphorylation levels in epithelial cells infected with Δ*Z1787* (119.13±28.76% of uninfected control, n = 4), relative to the wild-type parent strain, EHEC strain EDL 933 (4.70±5.19% of uninfected control, n = 4). Consistent with previous observations [20], EHEC strain CL56 (1.50±14.98%, n = 4), but not EPEC O127∶H6 strain 2348/69 (98.00±3.91%, n = 4), inhibited tyrosine phosphorylation of STAT-1 following IFNγ stimulation. However, complementation of the EHEC gene Z1787 knock out only partially restored pathogen inhibition of STAT-1 tyrosine phosphorylation (**Figure 6, Panel B**). Similarly, insertion of gene Z1787 on a pGEM-T vector into non-pathogenic *E. coli* strain HB101, did not result in the commensal being able to completely subvert STAT-1 tyrosine phosphorylation by IFNγ. In addition, transcript expression of Z1787 was not altered following EHEC growth in the presence of a polarized intestinal epithelial cell line (T84 cells), relative to pathogen growth in the absence of epithelial cells (**Supplemental** **Figure S3**). Collectively, these data indicate that gene Z1787 is not the only factor mediating EHEC subversion of STAT-1 activation. Instead, as for many other microbial pathogens, multiple factors likely contribute to pathogen subversion of immune signaling.

## Discussion

This is the first study to characterize gene expression changes of EHEC O157∶H7 grown in the presence of epithelial cells, compared to bacteria cultured in either minimal essential tissue culture medium (in the presence and absence of 5% CO_2_) or Penassay broth alone. The Affymterix *E. coli* 2.0 gene chip employed also was used by Hancock et al. [21] in their assessment of uropathogenic *E. coli* gene expression changes in biofilm growth versus planktonic conditions. An advantage of using this platform is that it contains the complete genomes of both pathogenic and non-pathogenic *E. coli*, thereby allowing for comparative studies and an extensive evaluation of expression patterns in both housekeeping genes and putative virulence factors. In our analyses, relative expression of housekeeping genes remained consistent, irrespective of EHEC growth conditions. By contrast, there was up-regulation in a number of virulence genes in response to exposure of the microbial pathogen to host epithelia. A putative protease, Z1787, encoded on OI (O-island)-50 [9], was identified by targeted gene disruption as a potential factor involved in EHEC subversion of IFNγ- Jak1,2-STAT-1 signaling.

Relatively few studies have evaluated gene expression changes of EHEC O157∶H7 in the context of an infection model. EHEC disease pathogenesis is initiated following pathogen contact with host epithelial cells, and subsequently results in microbial adhesion, toxin production, and modulation of host cell signal transduction cascades [3], [4]. Using microarrays, a previous study described differential gene expression profiles of EHEC O157∶H7 in response to bacterial growth in the presence of erythrocytes [22]. Down-regulation of 299 genes and up-regulation of 105 genes was reported following EHEC growth in the presence of rabbit erythrocytes, compared to culture of bacteria in minimal essential tissue culture medium alone [22]. Dahan et al. (2004) limited their study to gene expression changes of regulators and factors encoded by the locus of enterocyte effacement (LEE) pathogenicity island [30]. Common LEE encoded genes differentially regulated following exposure of EHEC to either erythrocytes or host epithelia include: *escR*, *escS*, *escT*, and *escU*. These results demonstrate reproducibility both within and between microarray studies. However, EHEC exposure to erythrocytes is not part of the infectious process, whereas EHEC exposure to epithelial cells lining the gut represents an early stage in disease pathogenesis [2].

Other studies employing microarray technology have evaluated gene expression profiling of *E. coli* in the context of phylogenetic studies [11]–[14], [31] and in response to either different environmental growth conditions or varying growth media [15]–[20]. For instance, one previous study assessed the effects of minimal glucose medium on uropathogenic *E. coli* gene expression, showing that 119 genes (2.8% of the genome) were up-regulated during pathogen growth in enriched medium, relative to the minimal glucose medium [32]. Two studies reported differential gene expression changes of urinary tract *E. coli* isolates during growth in biofilms, relative to planktonic growth [21], [26].

Previous studies characterizing gene expression changes for other enteric pathogens (*Salmonella* [33], *Shigella* [34], and *Listeria monocytogenes* [35]) identified virulence factors mediating microbial pathogenesis. These studies describe gene expression profiles of such pathogens as distinctly different during intracellular growth, relative to extracellular growth of the organism [33], [36]. Subsequent studies demonstrated genes encoding the expression of regulatory proteins and toxins during intracellular growth of the pathogens [37]. Such studies demonstrate that characterization of gene expression, by using microarray technology, can lead to a detailed description of pathogenic factors involved in disease progression [37].

Gene Z1787 encodes a putative protease and is present on OI-50 in EHEC O157∶H7 [7]. This bacterial protease may be released from EHEC during pathogen infection. Subsequent interactions of the protease with host epithelia could then contribute to EHEC subversion of STAT-1 tyrosine phosphorylation. Bacterial proteases facilitate pathogen infection by direct host tissue damage, by modulation of host cell signaling and by processing of other bacterial virulence factors [38]. For instance, the bacterial protease StcE, also encoded by EHEC O157∶H7, mediates bacterial adhesion [39] and displays proteolytic activity against both intestinal mucins and C1 esterase inhibitor [40]. StcE is insensitive to trypsin, chymotrypsin, and neutrophil elastase [40]. Another EHEC protease, EspP, is a serine protease that is cleaved and released into the extracellular environment during bacterial infection [41], which then potentially can destroy host cell surface receptors.

Bacterial proteases of other pathogens are also known to modulate signaling cascades and act as virulence factors. For instance, ScpC, a protease elaborated by *Streptococcus pyogenes* displays immune modulation during pathogen infection. During murine infection, ScpC degrades interleukin (IL)-8 and prevents neutrophil transmigration and activation. Multiple proteases of *Pseudomonas aeruginosa* (AprA, LasA, LasB, and protease IV) also are involved in disease pathogenesis. AprA, in particular, targets proteins of the complement system [42] and degrades pro-inflammatory cytokines [43]. LepA is another protease of *P. aeruginosa* that subverts NFκB activity by proteolytic activation of eukaryotic cell protease-activated receptors (PARs) [44]. Serralysin of *Serratia marcescens* is a protease that induces IL-6 and IL-8 expression in lung carcinoma cells. In addition, this protease activates NFκB via PAR-2 activation [45]. Taken together, these studies demonstrate that modulation (both activation and inhibition) of multiple host signal transduction cascades occurs in response to multiple bacterial proteases. Innate immune subversion can be mediated by bacterial proteolytic degradation of signaling molecules and their cognate receptors.

Gene expression analysis also should be validated using internal controls to represent consistent mRNA expression levels, regardless of bacterial growth conditions [24]. An evolutionary and phylogenetic analysis was previously used to identify seven housekeeping genes (*aroE*, *arcA*, *icd*, *mdh*, *mltD*, *pgi*, and *rpoS*) in *E. coli* O157∶H7 [25]. Among 21 EHEC isolates tested, these seven housekeeping genes displayed <8% variation at both the nucleotide and amino acid levels [25]. Multi-locus sequence typing of another set of seven housekeeping genes (*arcA*, *dnaE*, *mdh*, *gnd*, *gapA*, and *pgm*) showed phylogenetic relatedness among EHEC O157∶H7 isolates [46]. The *rfb*-*gnd* gene cluster also has been employed in evolutionary studies of EHEC O157∶H7 [47]. However, EHEC O157∶H7 housekeeping genes were not assessed in previous microarray studies. The results of the current study showed similar levels for both microarray signal intensities and transcript expression of the five housekeeping genes tested (*arcA*, *gapA*, *mdh*, *rfbA*, and *rpoS*) following EHEC growth in the presence and absence of epithelial cells.

In summary, this study is the first to report global gene expression changes following EHEC O157∶H7 growth in the presence and absence of host epithelia. We demonstrate increased gene expression of multiple potential EHEC virulence factors during bacterial growth in the presence of HEp-2 cells. Future studies need to be undertaken to better define the virulence potential and precise role of each of these genes in EHEC O157∶H7 disease pathogenesis.

## Materials and Methods

### Tissue culture

HEp-2 epithelial cells (American Type Culture Collection, Manassas, VA) were employed as a model epithelial cell line [4]. Briefly, HEp-2 cells were grown in Minimal Essential Medium containing 15% (v/v) fetal bovine serum, 2% (v/v) sodium bicarbonate, 2.5% (v/v) penicillin streptomycin, and 1% (v/v) fungizone (all from Invitrogen, Burlington, ON. Canada) [7]. Cells were grown in T25 flasks (Corning Incorporated, Corning, NY) at 37°C in 5% CO_2_ until confluent (2–3×10^6^). Confluent cells were trypsinized using 0.05% trypsin for 20 min at 37°C in 5% CO_2_. Trypsinized cells were then pelleted, centrifuged at 500 rpm for 5 min (Beckman Coulter, Mississauga, Ontario), resuspended in minimal essential tissue culture medium, and re-seeded into either T25 flasks or 6-cm Petri dishes (Becton Dickinson Labware, Franklin Lakes, NJ) and again grown at 37°C in 5% CO_2_ until confluent. Prior to bacterial infection, cells were incubated (24 hr at 37°C in 5% CO_2_) in minimal essential tissue culture medium without antibiotics and fetal bovine serum, as previously described [5].

A polarized intestinal epithelial cell line, T84, was employed as a complementary cell line for PCR experiments. As previously described [6], T84 cells were grown in a 1∶1 mixture of Dulbecco's modified Eagle's medium and Ham's F-12 medium. Culture medium was supplemented with 10% (vol/vol) fetal bovine serum (FBS), 2% penicillin-streptomycin, 2% sodium bicarbonate, and 0.6% L-glutamine (all obtained from Life Technologies, Grand Island, NY). Cells were grown to confluence and trypzinized with 0.25% trypsin.

### Bacterial strains and growth conditions

Bacterial strains used in this study included, enterohemorrhagic *E. coli* (EHEC), strains CL56 and EDL 933 (serotype O157∶H7) and enteropathogenic *E. coli* (EPEC) strain E2348/69 (serotype O127∶H6). EHEC strain CL56, originally isolated from a child with hemorrhagic colitis and hemolytic-uremic syndrome at the Hospital for Sick Children in Toronto, Canada is routinely used in our laboratory as a model EHEC strain [5]–[7]. The *eae*-positive EPEC strain E2348/69 was used for comparative purposes. Bacteria were grown, as previously described [5]–[7]. Briefly, strains were grown on 5% sheep blood agar plates (Becton, Dickinson and Company, Sparks, MD) at 37°C for 24 hr. Prior to infecting epithelial cells, bacteria were grown in 10 mL Penassay broth (Becton, Dickinson Co.) overnight at 37°C. For RNA extraction, overnight culture (1×10^9^ CFU per mL) of EHEC O157∶H7 was used to inoculate 10 mL of sterile Penassay broth (6 hr re-growth in 37°C), and 10 mL of minimal essential medium (6 hr re-growth in 37°C) incubated in either room air or 5% CO_2_ (6 hr re-growth in 37°C).

### Epithelial cell infection

EHEC O157∶H7 infection of HEp-2 or T84 epithelial cells was performed at a multiplicity of infection (MOI) of 100∶1 [6]. Briefly, 0.8 mL of an overnight bacterial culture was centrifuged at 3,000 rpm for 5 min, supernatants decanted, and bacterial pellets resuspended in 0.1 mL of Minimal Essential Medium. An aliquot of this bacterial resuspension (2×10^8^ CFU in 0.025 mL) was used to infect confluent epithelial cells grown in 6 cm Petri dishes. HEp-2 or T84 cells were infected with EHEC O157∶H7 for 6 hr at 37°C in 5% CO_2_. At the end of the 6 hr infection period, both adherent and non-adherent EHEC O157∶H7 cells were collected for total RNA extracts. For some experiments, at end of the 6 hr infection period, cells were washed and then stimulated with IFNγ (50 ng/mL for 0.5 hr at 37°C in 5% CO_2_), followed by whole cell protein extracts for immunoblotting.

### RNA extraction

RNA extracts of EHEC O157∶H7 from each of the four growth conditions were prepared using the Qiagen RNeasy mini-prep kit (Qiagen, Mississauga, Ontario, Canada). A total of 19 RNA extracts (that is, 4 or 5 gene chips for each of the four growth conditions) were prepared. Briefly, bacteria were harvested at 5,000 rpm for 5 min at 4°C. Bacterial pellets were re-suspended in 0.1 mL Tris-EDTA buffer (pH 7.6; Fisher Scientific, Ottawa, Ontario, Canada) containing 0.04 mg Lysozyme (Pierce, Rockford, IL) at room temperature for 3–5 min. Subsequently, 0.350 mL of lysis buffer (RLT buffer; Qiagen) containing 1% β-mercaptoethanol (Kodak) was added and bacterial re-suspensions vortexed vigorously. The entire sample was then loaded into a mini spin column (QIA shredder; Qiagen) and centrifuged in a mini-centrifuge (Beckman Coulter, Mississauga, Ontario, Canada) for 2 min. Supernatants were collected, washed with 0.25 mL of 100% ethanol, and loaded into a silica membrane mini-column (RNeasy mini column; Qiagen). Samples were again centrifuged at 10,000 rpm for 15 sec, flow throughs discarded, and 0.350 mL of wash buffer (RWI buffer; Qiagen) added to each mini-column. Ethanol-containing wash buffer (RPE buffer; Qiagen) was then loaded into each mini-column and centrifuged at 10,000 rpm for 1 min. Purified RNA was collected off the silica membrane using 0.025 mL of RNase free water (Qiagen) and centrifugation at 10,000 rpm for 1 min. DNA was removed by adding 0.80 mL (27.3 Kunitz units) DNase in DNA digest buffer (Qiagen) for 15 min at room temperature.

### RNA concentration and purity

Purified RNA was diluted (1/10) in DEPC-treated water (Fischer Scientific) and OD 260/280 readings were obtained using a spectrophotometer (Ultrospec 2100 Pro UV–VIS spectrophotometer; Biochrom, Cambridge, England). Samples with an OD 260/280 ratio of 1.8 or greater and a minimum concentration of 0.5 mg/mL were used for subsequent analyses. RNA quality was determined on a 1% agarose gel (Sigma Aldrich). One µg of each RNA sample was combined with loading buffer, and gels subject to electrophoresis at 80 V for 40–50 min at room temperature. As a standard, a high DNA mass ladder (Invitrogen) was added to the first lane of the agarose gel. RNA bands were visualized under an ultraviolet lamp.

### Microarray

For microarray analysis, high quality RNA was sent to a microarray facility (The Centre for Applied Genomics, Hospital for Sick Children, Toronto, Ontario, Canada). An independent internal RNA quality control was performed using the Agilent 2100 bioanalyzer (Aqilent Technologies Inc., Santa Clara, CA). Subsequently, RNA extracts were processed for microarray analysis, as previously described [21]. Detailed protocols describing labeling, hybridization, image acquisition, and normalization can be obtained from: www.affymetrix.com. The *E. coli* Genome 2.0 gene chip (Affymetrix, Santa Clara, CA), which contains the complete genomes of four *E. coli* strains (non-pathogenic *E. coli* K12, strain MG1655, uropathogenic *E. coli*, strain CFT073, and enterohemorrhagic *E. coli* O157∶H7, strains EDL 933 and Sakai) was employed. The gene chip consists of 10,000 probe sets for all 20,366 genes of the four bacteria. The number of probe sets unique to EDL 933 O157∶H7 include: 5,480 while the number of EDL 933 O157∶H7-specific transcripts is 5,376 (www.affymetrix.com).

### Microarray data analyses

Data analyses from 19 gene chips were performed using the Partek Genomics Suite version 6.08.0128 (Partek Inc., St Louis, MO) (GEO Accession# GSE14238). Affymetrix .cel files were imported into Partek with the Robust Multichip Average algorithm (RMA; [48]) feature ‘on’ in order to normalize data sets. The normalization feature was employed to perform background correction, quantile normalization across gene chips, log base 2 transformation, and median polish summarization. Data were filtered to include only EHEC O157∶H7 strains EDL 933 and Sakai specific genes. The Partek workflow was set to ‘Gene Expression’ for subsequent data analyses.

### Statistics

One-way analysis of variance (ANOVA) was performed to identify genes altered by at least a 2.0-fold increase or decrease in expression between groups. The numbers of significantly up- and down-regulated genes in each comparison group were identified by applying a *p* value of <0.05, together with fold change cut-offs of 2.0-fold decrease and 2.0-fold increase [6], [21]–[22].

### cDNA synthesis

For PCR analysis, cDNA synthesis was performed using the Invitrogen Thermoscript kit (Invitrogen). Briefly, 1 µg of RNA template was reverse transcribed using random hexamer primers in buffered conditions (1× synthesis buffer; 0.1 M DTT, 40 U/µl RNaseOUT™; 15 U/µl Thermoscript™RT). The reaction was incubated for 50 min at 60°C and terminated at 85°C for 5 min. At the end of the cycle, 200 U RNase H (Invitrogen) was added to each reaction tube and samples incubated at 37°C for 20 min and then stored at −20°C.

### Reverse Transcription PCR (RT-PCR)

The cDNA templates were amplified using 0.2 µM of each specific primer (**Table 2**), 0.2 mM dNTP, 25 mM MgCl_2_, 1.25 U *Taq* polymerase (Fermentas, Burlington, Ontario, Canada) in supplied buffer (10 mM Tris-HCl, pH 8.8; 50 mM KCl, 0.08% NP40). The reaction was incubated at 95°C for 10 min, followed by 45 cycles of 95°C (30 sec), 55°C (60 sec) and 72°C (30 sec). All products were resolved on a 2% agarose gel in 1× TAE buffer and then stained with ethidium bromide (50 µg/mL) for visualization of bands.

**Table 2:** Primers pairs used in this study for PCR.

Gene	Primer Pairs	Reference
*arcA*	F: 5′-GAA GAC GAG TTG GTA ACA CG-3′	[25]; this study
	R: 5′-CTT CCA GAT CAC CGC AGA AGC-3	
*gapA*	F: 5′-GAT TAC ATG GCA TAC ATG CTG-3′	[49]; this study
	R: 5′-CAG ACG AAC GGT CAG GTC AA-3′	
*mdh*	F: 5′-CAA CTG CCT TCA GGT TCA GAA-3′	[25]; this study
	R: 5′-GCG TTC TGG ATG CGT TTG GT-3′	
*rfbA*	F: 5′-GCG CTT TCG ACA TGT TGG ACA CTT-3′	[50]; this study
	R: 5′-AAT TCC GTT CTT CCC TGG GTG CTA-3′	
*rpoS*	F: 5′-TAT GAG TCA GAA TAC GCT GAA A-3′	[25]; this study
	R: 5′-GGA ACA GCG CTT CGA TAT TCA G-3′	
Z1787	F:5′-ACT GTC ACT GTC AAC TCT CAG-3′	this study
	R:5′-GGC AAC CAC TCA GGA AAA TG-3′	

### Quantitative real time polymerase chain reaction (qRT-PCR)

Transcript expression of EHEC O157∶H7 grown in the presence or absence of epithelial cells were compared using gene-specific primers. A series of 10-fold dilutions of cDNA was used to generate a standard curve and a 1∶50 dilution of sample cDNA quantified, relative to this series. The qRT-PCR reactions were performed using either the ABI 7900HT Real-Time PCR System (Applied Biosystems) or the BioRad CFX96 (BioRad Laboratories, Hercules, CA) and SYBR Green PCR Master Mix (Applied Biosystems), in triplicate. Primers were obtained from IDT DNA (Integrated DNA Technologies Inc., Coralville, IA) and constructed under HPLC conditions. The qRT-PCR reactions were performed in a 20 µl reaction with 0.2 µM primer. Template was pre-incubated at 50°C for 2 min, denatured at 95°C for 10 min and subjected to 45 cycles of the following thermal conditions: 95°C (15 sec) and 60°C (60 sec). The product cycle threshold (CT) was determined from ROX-normalized fluorescence emission and used to calculate the initial input of template. All values reported represent the mean of at least three independent experiments.

### Gene mutation

Isogenic mutants of EHEC O157∶H7, strain EDL 933 were constructed using the Lambda Red technique [28]–[29]. Plasmids used for recombineering included pKD46 (AmpR; Red recombinase expression plasmid) and pKD4 (template plasmid with FLP recognition target sites flanking a kanamycin resistance gene). Primers designed for the construction of PCR-generated DNA fragments were: 5′-CAA TCT TCA TCA TTA AAT AAA AGG AGT GCT TAT GGT GTA GGC TGG AGC TGC TTC G-3′ (forward primer) and 5′-GTC TTA TAA ATT TAA AAC CAT AGA AAA AAT CAA TTA TGA TAT GAA TAT CCT CCT TA-3′ (reverse primer). PCR products were generated by using primers designed to include ends of the gene that was excised as well as flanking regions of the gene. The extensions attached to forward and reverse primers included, 3′-GTG TAG GCT GGA GCT GCT TCG-5′ and 3′- ATA TGA ATA TCC TCC TTA-5′, respectively. Plasmid DNA was extracted by using a Miniprep Kit (Qiagen). Plasmid pKD4 was used as template for the primer pair to amplify DNA fragments for recombination. PCR amplicons were verified on a 1% agarose gel and subsequently purified using a PCR Purification Kit (Qiagen).

EHEC O157∶H7 strain EDL933 was transformed with pKD46 using a standard calcium chloride method. These transformants containing the Red helper plasmid pKD46 were made electrocompetent by growing 30 ml cultures with ampicillin and L-arabinose at 30°C on a shaker until mid-log phase when OD 600 reached 0.6. The cells were then pelleted by centrifugation at 8,000 rpm for 10 min at 4°C, washed 3 times with 25 mL ice cold water, and resuspended in 100 µL ice cold water.

Purified PCR generated DNA was electroporated into electrocompetent EDL 933 transformants containing pKD46-AmpR. Electroporation was performed using the Gene Pulser Xcell total system (BioRad). Following electroporation, cells were recovered using 1 mL Super Optimal Catabolite (Invitrogen) medium at 30°C for 3 hours. Mutants were then plated onto LB-Kan (25 µg/mL) agar plates and grown overnight at 42°C. Colonies were screened using PCR primers for the gene sequence of the target gene to verify loss of the gene. New flanking junctions in the mutants were also verified by PCR.

Complementation was performed as follows: PCR products were amplified and purified, as described above. pGEM-T vector ligation reactions were then performed using the pGEM-T kit (Promega, Madison, WI) with 2× buffer (5 µL), pGEM-T (1 µL), PCR product (1.5 µL of 61.2 µg), and T4 DNA ligase (1 µL) up to a volume of 10 µL. Reactions were left at 4°C for 24 hours. Competent bacteria were generated, as described above, with pelleted cells re-suspended using 10 mL ice-cold calcium chloride (0.1 M). Re-suspended bacteria were incubated on ice for 10 min, centrifuged at 4,000 rpm at 4°C for 10 min, and supernatants decanted. Bacterial pellets were then resuspended to a final volume of 50 µL in calcium chloride (0.1 M). Competent bacteria were then transformed with the pGEM-T ligation products: 2 µL of each ligation reaction was added to 50 µL of bacteria and placed on ice for 30 min. Cells were then heat shocked for 2 min in a water bath at 42°C. Immediately after, cells were returned to an ice-water bath for 2 min. Bacteria transformed with the ligation reaction were then recovered using 250 µL S.O.C. medium at room temperature and incubated at 37°C for 1.5 hrs on a shaker. Transformants were then plated onto LB/ampicillin/X-Gal plates, which were incubated at 37°C overnight.

### Whole cell protein extracts and immunoblotting

Whole cell protein extracts were conducted, as previously described [5]. Briefly, cell extracts were centrifuged at 13,000 rpm for 20 sec at 20°C, supernatants decanted and pellets re-suspended in 0.150 mL RIPA buffer (1% Nonidet P-40, 0.5% sodium deoxylate, 0.1% sodium dodecyl sulfate [SDS] in PBS) supplemented with 150 mM NaCl, 50 mM sodium fluoride, 1 mM sodium ortho-vanadate, 20 µg/mL phenylmethylsulfonyl fluoride, 15 µg/mL aprotinin, 2 µg/mL leupeptin, and 2 µg/mL pepstatin A (all from Sigma Aldrich, Oakville, Ontario, Canada), vortexed well, and left on ice for 20 min. Re-suspended pellets were centrifuged again at 12,000 rpm for 10 min at 4°C and supernatants stored at −80°C until further analysis by immunoblotting.

Immunoblotting was conducted, as previously described [5]. Whole cell protein extracts were combined with SDS-PAGE loading buffer in a 2∶1 (v/v) ratio, samples boiled for 3 min, and then loaded into precast 10% polyacrylamide gels (Ready Gel®; BioRad Laboratories, Hercules, CA). Gels were electrophoresed at 150 V for 1 hr at room temperature followed by protein transfer onto nitrocellulose membranes (BioTrace NT; Pall Corporation, Ann Arbor, MI) at 110 V for 1.5–2 hr at 4°C.

Membranes were incubated in Odyssey blocking buffer (LICOR Biosciences, Lincoln, NE) for 1 hr at 20°C on a shaker followed by incubation with primary antibodies (4°C overnight on a shaker). Primary antibodies included: anti-latent-STAT-1 (1 in 5,000 dilution; Santa Cruz Biotechnologies, Santa Cruz, CA), anti-phospho-STAT-1 (1 in 1,000 dilution; Cell Signaling, Beverly, MA), and anti-β-actin (1 in 5,000 dilution; Sigma). Membranes were washed 4 times with PBS+ 0.1% Tween (5 min per wash) and then incubated with secondary antibodies (1 hr at 4°C on a shaker). Secondary antibodies included: IRDye 800 goat anti-rabbit IgG (1 in 20,000 dilution; Rockland Immunochemicals, Gilbertsville, PA) and Alexa Fluor® 680 goat anti-mouse IgG (1 in 20,000 dilution; Molecular Probes, Eugene, OR) [5], [6].

Immunoblots were scanned into an infrared imaging system (Odyssey, LI-COR Biosciences), with both the 700 nm and 800 nm channels ‘on’ and immunoblots scanned at a resolution of 169 µm [5]. Using automated software (LI-COR Biosciences) densitometry was performed to obtain the integrative intensity of all positively staining bands. Integrative intensity values for each of the phospho-STAT-1 and latent-STAT-1 bands were normalized to the integrative intensity values obtained for the corresponding anti-β-actin bands. Uninfected cells stimulated with IFNγ were used as positive controls (standardized to 100%). Densitometry values obtained from EHEC O157∶H7 infected samples were then calculated as a percentage of the positive, uninfected control [5].

## Supporting Information

Figure S1 Principle component analysis (PCA) of microarray chips: Seventeen separate samples generated a PCA value of 68.4%. Each point represents an array chip: circles represent EHEC O157∶H7, strain CL56 grown in the presence of HEp-2 cells; triangles represent bacteria grown in minimal essential medium in 5% CO_2_; diamonds represents the pathogen grown in minimal essential medium alone and squares represent organisms grown in Penassay broth alone.
(0.04 MB PDF)

Figure S2 Volcano plots show differential expression of EHEC O157∶H7, strain CL56 genes under three individual growth conditions. Data points were extracted from a 1-way analysis of variance (ANOVA): [Panel A] Comparison of genes with altered expression between EHEC grown in the presence of epithelial cells versus bacteria grown in minimal essential medium in 5% CO_2_; [Panel B] Bacterial growth in the presence of epithelial cells versus bacterial growth in tissue culture medium in room air; [Panel C] Pathogen growth in the presence of HEp-2 cells, compared with growth in Penassay broth. . The x-axis represents ‘log fold change’ and the corresponding dark vertical lines represent cut-offs at log 2.0-fold decreases and increases. The y-axis represents p-values and the corresponding. Values presented represent the number of down- and up-regulated genes, respectively. Top 20 up-regulated genes for each panel are shown in Supplemental **Tables S1** **,** **S2** **and** **S3**, respectively.
(0.46 MB PDF)

Figure S3 Relative expression patterns of EHEC O157∶H7, strain CL56 gene Z1787 and *gapA*, respectively. qRT-PCR of Z1787 showing transcript expression for EHEC O157∶H7 under varying growth conditions. Data points are derived from triplicates of EHEC O157∶H7 grown in the presence or absence of polarized epithelial cells, T84. Data analysis was performed using the BioRad1.1CFXManager. Black bars represent EHEC O157∶ H7 grown in the presence of T84 cells; stripped bars represent the pathogen grown in minimal essential medium in 5% CO_2_; grey bars represent microbial growth in LB broth in 5% CO_2_; white bars represent bacterial growth in LB broth in standard conditions. Y-axis scale bar adjusted to be consistent with Figure 4, Panel B.
(0.03 MB PDF)

Table S1 (0.01 MB DOC)

Table S2 (0.01 MB DOC)

Table S3 (0.01 MB DOC)
