# Supplementary figures and images for: Enterohemorrhagic Escherichia coli O157∶H7 Gene Expression Profiling in Response to Growth in the Presence of Host Epithelia
Source: PLoS One. 2009 Mar 18;4(3):e4889. doi: 10.1371/journal.pone.0004889 (PMC2654852; doi:10.1371/journal.pone.0004889)

**Supplemental FIGURE S1:**

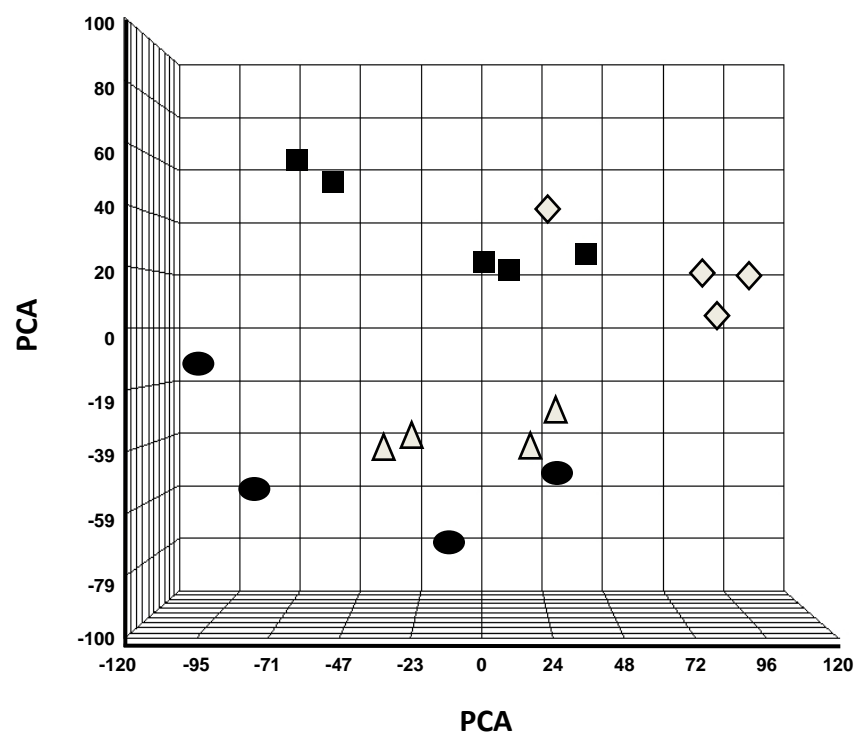

Supplement: Figure S1 — Principle component analysis (PCA) of microarray chips: Seventeen separate samples generated a PCA value of 68.4%. Each point represents an array chip: circles represent EHEC O157∶H7, strain CL56 grown in the presence of HEp-2 cells; triangles represent bacteria grown in minimal essential medium in 5% CO2; diamonds represents the pathogen grown in minimal essential medium alone and squares represent organisms grown in Penassay broth alone. (0.04 MB PDF) [file pone.0004889.s001.pdf]

**Supplemental FIGURE S2:**

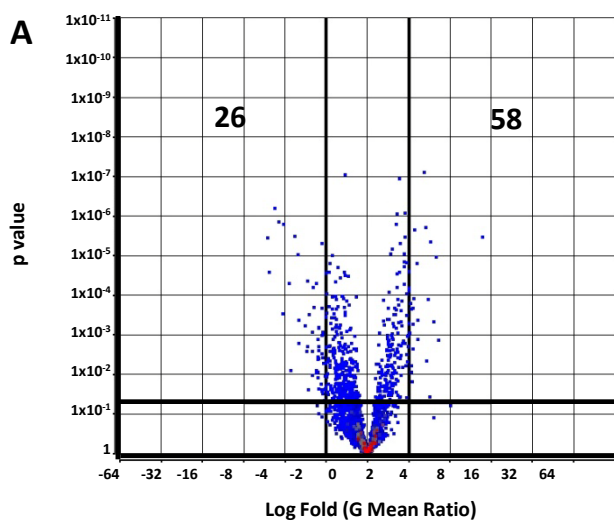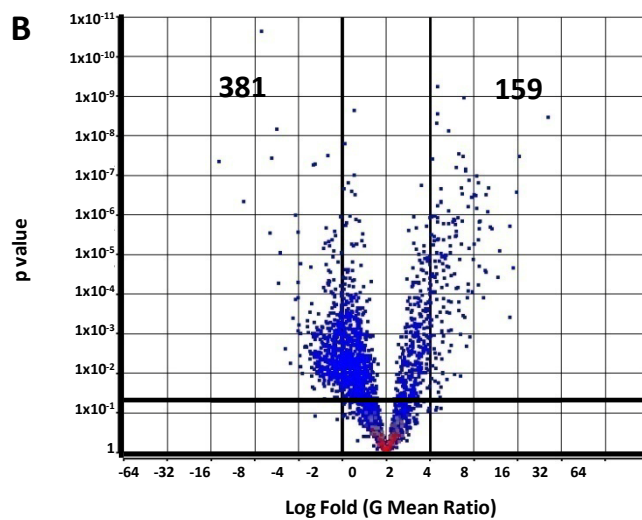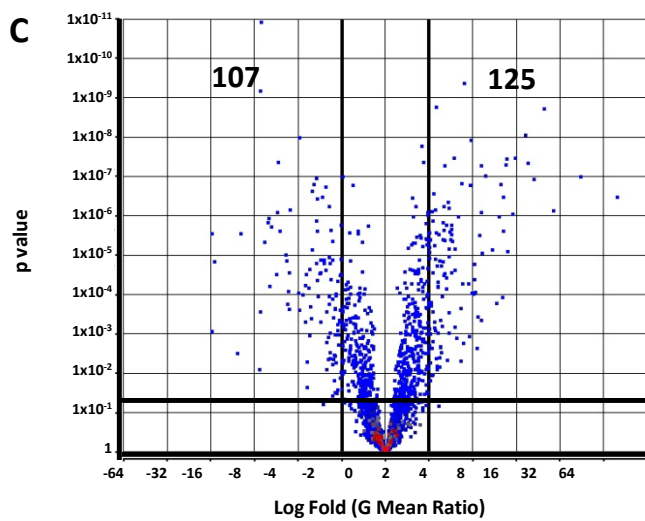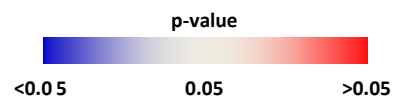

Supplement: Figure S2 — Volcano plots show differential expression of EHEC O157∶H7, strain CL56 genes under three individual growth conditions. Data points were extracted from a 1-way analysis of variance (ANOVA): [Panel A] Comparison of genes with altered expression between EHEC grown in the presence of epithelial cells versus bacteria grown in minimal essential medium in 5% CO2; [Panel B] Bacterial growth in the presence of epithelial cells versus bacterial growth in tissue culture medium in room air; [Panel C] Pathogen growth in the presence of HEp-2 cells, compared with growth in Penassay broth. . The x-axis represents ‘log fold change’ and the corresponding dark vertical lines represent cut-offs at log 2.0-fold decreases and increases. The y-axis represents p-values and the corresponding. Values presented represent the number of down- and up-regulated genes, respectively. Top 20 up-regulated genes for each panel are shown in Supplemental Tables S1 , S2 and S3 , respectively. (0.46 MB PDF) [file pone.0004889.s002.pdf]

**Supplemental FIGURE S.3:**

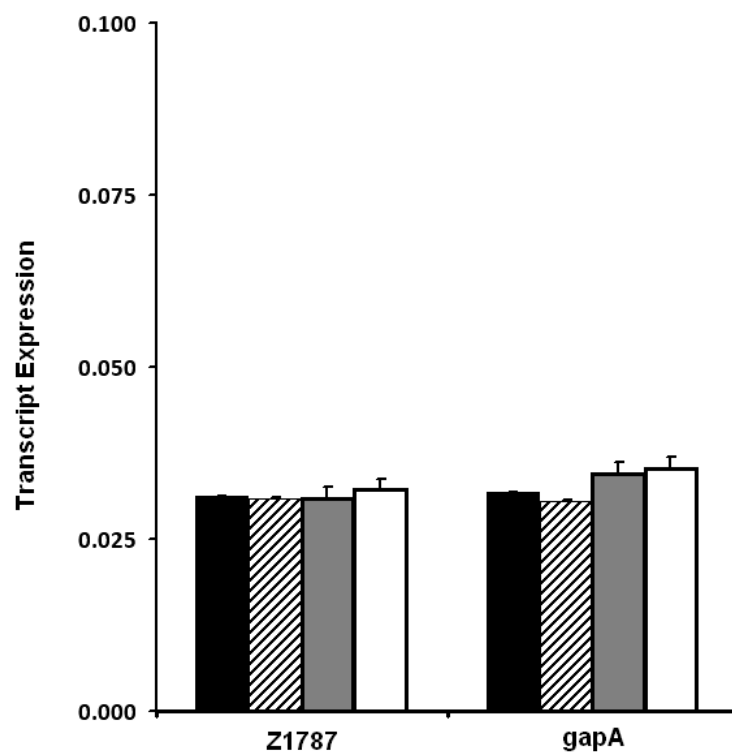

Supplement: Figure S3 — Relative expression patterns of EHEC O157∶H7, strain CL56 gene Z1787 and gapA, respectively. qRT-PCR of Z1787 showing transcript expression for EHEC O157∶H7 under varying growth conditions. Data points are derived from triplicates of EHEC O157∶H7 grown in the presence or absence of polarized epithelial cells, T84. Data analysis was performed using the BioRad1.1CFXManager. Black bars represent EHEC O157∶ H7 grown in the presence of T84 cells; stripped bars represent the pathogen grown in minimal essential medium in 5% CO2; grey bars represent microbial growth in LB broth in 5% CO2; white bars represent bacterial growth in LB broth in standard conditions. Y-axis scale bar adjusted to be consistent with Figure 4, Panel B. (0.03 MB PDF) [file pone.0004889.s003.pdf]
