# Supplementary material for: Enterohemorrhagic Escherichia coli O157∶H7 Gene Expression Profiling in Response to Growth in the Presence of Host Epithelia
Source: PLoS One. 2009 Mar 18;4(3):e4889. doi: 10.1371/journal.pone.0004889 (PMC2654852; doi:10.1371/journal.pone.0004889)
Supplement: Table S1 — (0.01 MB DOC) [file pone.0004889.s004.doc]

**Supplemental TABLE S1: Top 20 up-regulated genes showing the greatest fold increase between EHEC O157:H7 grown in the presence of cells, relative to growth in culture medium in 5% CO_2_.**

| **Probeset ID** | **Gene** | **Gene product / functional description** | **Fold change** |
| --- | --- | --- | --- |
| 1767702_s_at | *ilvC* | ketol-acid reductoisomerase | 6.94 |
| 1762060_s_at | *guaB* | IMP dehydrogenase | 3.32 |
| 1766583_s_at | *aceF* | pyruvate dehydrogenase | 3.19 |
| 1761408_s_at | *entD* | enterobactin synthetase component D | 3.05 |
| 1763215_s_at | *bioD* | dethiobiotin synthetase | 2.89 |
| 1762024_s_at | ECs5032 | hypothetical protein / unknown function | 2.85 |
| 1761688_s_at | *ydgR* | putative transport protein | 2.76 |
| 1762165_s_at | *bioC* | biotin biosynthesis; reaction prior to pimeloyl CoA | 2.68 |
| 1767691_s_at | *gltB* | glutamate synthase, large subunit | 2.65 |
| 1759270_s_at | *gltD* | glutamate synthase, small subunit | 2.60 |
| 1767820_s_at | *dniR* | transcriptional regulator for nitrite reductase | 2.33 |
| 1761728_s_at | Z5129 | hypothetical protein / unknown function | 2.32 |
| 1759701_s_at | *Uup* | putative ATP-binding component of a transport system | 2.30 |
| 1763916_s_at | *recG* | DNA helicase | 2.20 |
| 1765592_s_at | *urge* | putative urease accessory protein G | 2.17 |
| 1761091_s_at | Z5128 | hypothetical protein / unknown function | 2.16 |
| 1769216_s_at | *ycaO* | hypothetical protein / unknown function | 2.15 |
| 1767859_s_at | *ordL* | probable oxidoreductase | 2.14 |
| 1767609_s_at | *escU* | EscU | 2.11 |
| 1761586_s_at | *leuB* | 3-isopropylmalate dehydrogenase | 2.09 |
