# Supplementary material for: Enterohemorrhagic Escherichia coli O157∶H7 Gene Expression Profiling in Response to Growth in the Presence of Host Epithelia
Source: PLoS One. 2009 Mar 18;4(3):e4889. doi: 10.1371/journal.pone.0004889 (PMC2654852; doi:10.1371/journal.pone.0004889)
Supplement: Table S2 — (0.01 MB DOC) [file pone.0004889.s005.doc]

**Supplemental TABLE S2: Top 20 up-regulated genes showing the greatest fold increase between EHEC O157:H7 grown in the presence of cells, relative to growth in culture medium alone.**

| **Probeset ID** | **Gene** | **Gene product / functional description** | **Fold change** |
| --- | --- | --- | --- |
| 1765419_s_at | ECs2526 | Hypothetical protein / unknown function | 12.88 |
| 1765359_s_at | *chuW* | putative oxygen independent coproporphyrinogen III oxidase | 8.19 |
| 1766483_s_at | Z1178 | putative receptor | 7.82 |
| 1763313_s_at | *chuS* | putative hemehemoglobin transport protein | 7.41 |
| 1761408_s_at | *entD* | enterobactin synthetase component D | 7.05 |
| 1762260_s_at | *chuA* | outer membrane hemehemoglobin receptor | 7.00 |
| 1767702_s_at | *ilvC* | ketol-acid reductoisomerase | 5.95 |
| 1762165_s_at | *bioC* | biotin biosynthesis; reaction prior to pimeloyl CoA | 5.80 |
| 1767505_s_at | *entE* | 2,3-dihydroxybenzoate-AMP ligase | 5.33 |
| 1763001_s_at | *terB* | putative phage inhibition, colicin resis. & tellurite resis. | 5.17 |
| 1760999_s_at | *wbdP* | glycosyl transferase | 5.00 |
| 1761302_s_at | *terC* | putative phage inhibition, colicin resis. & tellurite resis. | 4.93 |
| 1764929_s_at | *nuoG* | NADH dehydrogenase I chain G | 4.81 |
| 1766799_s_at | *yafH* | putative acyl-CoA dehydrogenase (EC 1.3.99.-) | 4.81 |
| 1763337_s_at | *entF* | ATP-dependent serine activating enzyme | 4.79 |
| 1759275_at | ECs1567 | Hypothetical protein / unknown function | 4.64 |
| 1769044_s_at | *chuY* | Hypothetical protein / unknown function | 4.40 |
| 1761586_s_at | *leuB* | 3-isopropylmalate dehydrogenase | 4.35 |
| 1761631_s_at | *terD* | putative phage inhibition, colicin resis. & tellurite resis. | 4.35 |
| 1769094_s_at | Z2150 | Hypothetical protein / unknown function | 4.34 |
