# Supplementary material for: Enterohemorrhagic Escherichia coli O157∶H7 Gene Expression Profiling in Response to Growth in the Presence of Host Epithelia
Source: PLoS One. 2009 Mar 18;4(3):e4889. doi: 10.1371/journal.pone.0004889 (PMC2654852; doi:10.1371/journal.pone.0004889)
Supplement: Table S3 — (0.01 MB DOC) [file pone.0004889.s006.doc]

**Supplemental TABLE S3: Top 20 up-regulated genes showing the greatest fold increase between EHEC O157:H7 grown in the presence of cells, relative to growth in Penassay broth.**

| **Probeset ID** | **Gene** | **Gene product / functional description** | **Fold change** |
| --- | --- | --- | --- |
| 1762260_s_at | *chuA* | outer membrane hemehemoglobin receptor | 39.84 |
| 1763313_s_at | *chuS* | putative hemehemoglobin transport protein | 22.25 |
| 1768770_s_at | *chuT* | putative periplasmic binding protein | 14.55 |
| 1765359_s_at | *chuW* | putative oxygen independent coproporphyrinogen III oxidase | 12.50 |
| 1761408_s_at | *entD* | enterobactin synthetase component D | 10.63 |
| 1766483_s_at | Z0726 | Hypothetical protein / unknown function | 9.59 |
| 1765419_s_at | ECs2526 | Hypothetical protein / unknown function | 9.30 |
| 1760730_s_at | *chuU* | putative permease of iron compound ABC transport system | 7.86 |
| 1763337_s_at | *entF* | ATP-dependent serine activating enzyme | 7.61 |
| 1762165_s_at | *bioC* | biotin biosynthesis; reaction prior to pimeloyl CoA | 6.95 |
| 1769044_s_at | *chuY* | Hypothetical protein / unknown function | 6.92 |
| 1766687_s_at | Z4919 | putative ATP-binding protein of ABC transport system | 6.81 |
| 1767505_s_at | *entE* | 2,3-dihydroxybenzoate-AMP ligase | 6.53 |
| 1765174_s_at | Z4912 | Hypothetical protein / unknown function | 6.52 |
| 1765765_s_at | Z1178 | putative receptor | 6.45 |
| 1768228_s_at | *actP* | acetate permease | 6.30 |
| 1759808_s_at | *fadB* | 4-enzyme protein | 6.06 |
| 1768186_s_at | *fhuF* | Hypothetical protein / unknown function | 5.85 |
| 1766799_s_at | *yafH* | putative acyl-CoA dehydrogenase (EC 1.3.99.-) | 5.49 |
| 1761010_s_at | *Beta* | choline dehydrogenase, a flavoprotein | 4.94 |
